# IgG expressed by renal tubular epithelial cells in epithelial mesenchymal transformation and interstitial fibrosis in diabetic kidney disease

**DOI:** 10.1080/0886022X.2025.2458764

**Published:** 2025-02-03

**Authors:** Xinyao Wang, Zhenling Deng, Yue Wang

**Affiliations:** Department of Nephrology, Peking University Third Hospital, Beijing, China

**Keywords:** Diabetic kidney disease, epithelial mesenchymal transition, immunoglobulin G, renal tubular epithelial cell, renal tubulointerstitial fibrosis

## Abstract

Studies have reported that immunoglobulin G (IgG) "deposited" in the basement membrane of renal tubules is associated with tubulointerstitial damage in patients with diabetic kidney disease (DKD). Our previous study found that renal tubular epithelial cells (RTECs) can express and secrete IgG (RTEC-IgG) which may be associated with fibrosis. The present study aimed to explore the role of RTEC-IgG in renal tubulointerstitial fibrosis (TIF) in DKD. The results showed that RTEC-IgG expression was up-regulated in the renal tubulointerstitium of DKD patients and was associated with worse kidney function, more severe anemia, and higher interstitial fibrosis and tubular atrophy (IFTA) scores, and positively correlated with tubular epithelial mesenchymal transition (EMT) and TIF. IgG expression was also enhanced in the renal tubulointerstitium of DKD mice, which was positively correlated with TIF. High glucose induced an over expression of IgG in human renal tubular epithelial cells, and knockdown of IgG with siRNA relieved the expression of α-smooth muscle actin (SMA), collagen IV, fibronectin, and transforming growth factor (TGF)-β1 under high glucose conditions. In conclusion, our study suggests that RTEC-IgG is involved in the development of DKD by promoting EMT and interstitial fibrosis via TGF-β1.

## Introduction

Diabetic kidney disease (DKD) is a common microvascular complication of diabetes mellitus and a major cause of end-stage renal disease (ESRD). DKD was once recognized as a glomerular disease, with early pathology manifesting as thickening of the glomerular basement membrane, followed by mesangial matrix expansion, development of K–W nodules and glomerulosclerosis. However, an increasing number of studies have shown that tubulointerstitial fibrosis (TIF), characterized by excessive deposition of extracellular matrix (ECM) such as collagen (Col) and fibronectin (FN) in the renal tubulointerstitium [1], is more associated with decreased kidney function and poor kidney prognosis [2]. Epithelial mesenchymal transition (EMT), characterized by a loss of tight junctions between epithelial cells with deletion of expression of the epithelial cell marker E-cadherin, followed by the acquisition of mesenchymal cell phenotypes such as α-smooth muscle actin (α-SMA), Vimentin and the expression of ECM such as Col, FN and others [3], transform renal tubular epithelial cells (RTECs) into myofibroblasts, eventually resulting in TIF [1]. Activation of the transforming growth factor (TGF)-β1 signaling pathway plays key role in EMT and TIF [4, 5].

There are studies demonstrated that in kidney biopsy tissues from DKD patients ‘deposited’ immunoglobulin G (IgG) in the glomerular and tubular basement membranes, which is associated with kidney injury and poor prognosis [6, 7], with the ‘deposited’ IgG along tubular basement membrane (TBM) more associated with proteinuria and severity of tubulointerstitial damage [8]. At the same time, mounting evidence have confirmed the expression of Immunoglobulins (non-B-Igs) in tissue cells including epithelial cells [9–11], cardiomyocytes [12, 13], hematopoietic cells [14], myeloid cells [15], and even in immune-privileged cells such as neurons [16], spermatogenic cells [17] and intraocular epithelium and endothelium [18], and non-B-Igs are physiologically involved in immune defense [10, 11], cytoskeletal and extracellular matrix protein formation [19], whereas pathologically are involved in malignant tumors [9, 20–22], autoimmune diseases and chronic inflammatory [23], providing with a new perspective to better understand not only basic immunology but also various Ig-related diseases. Specifically, non-B-IgG is involved in EMT and tissue fibrosis, Peng found that overexpression of IgG by salivary-like cystic carcinoma cells could induce EMT to promote tumor invasion and metastasis [22], and Yu reported that IgG by adipose cells could induce fibrosis through the TGF-β/SMAD pathway [24].

Our previous research showed that single cell of human glomerular mesangial cells [25], podocytes [26], tubular epithelial cells [27] has gene transcription of multiple immunoglobulin including IgG, and that *in vitro* cultured glomerular mesangial cells can express IgA with IgA1 as the majority [25], podocytes can express IgG with IgG4 as the majority [26], and renal tubular epithelial cell line HK-2 can express and secrete IgG [28]^.^ The antibody RP215 specifically recognizes glycosylation epitopes of IgG heavy chains expressed by human non-B cells and is widely used in the study of IgG expressed by human non-B cells [20, 29]. Our pilot study confirmed that renal tubular epithelial cells in patients with DKD can express IgG (RTEC-IgG).

Despite the use of drugs such as renin–angiotensin–aldosterone (RAAS) inhibitors and sodium-glucose co-transporter-2 (SGLT-2) inhibitors to improve the symptoms and prognosis of DKD in recent years [30], DKD still accounts for half of the global burden of ESRD [31]. This study was to investigate the relationship between RTEC-IgG and tubular EMT and TIF as well as clinical features in patients with DKD, providing a new perspective on DKD pathophysiology and potential therapeutic targets.

## Materials and methods

### Patients

This study reviewed 60 patients with type 2 diabetes mellitus(T2DM) who underwent kidney biopsy and pathologically confirmed DKD from January 2014 to January 2021 at the Third Hospital of Peking University. Inclusion criteria were (1) age > 18 years; (2) clinically diagnosed T2DM; (3) DKD confirmed by kidney biopsy, and (4) eGFR > 15 mL/min/1.73m^2^. The exclusion criteria were (1) coexistence with nondiabetic kidney disease, such as primary glomerular disease like IgA nephropathy, membranous nephropathy, or comorbid systemic disease like systemic lupus erythematosus, vasculitis, etc., and (2) hormones or immunosuppressants had been applied at the time of kidney biopsy. Kidney biopsy is indicated in patients with T2DM with kidney damage, including acute kidney injury, a significant short-term increase in proteinuria, or the presence of hematuria of glomerular origin. At least two nephrologists or kidney pathologists re-diagnosed DKD and re-confirmed pathologic classification by interpretation of kidney specimens according to Tervaert’ s classification [32]. Complete clinical information about the patients at the time of kidney biopsy was collected, including age, sex, kidney function, urinary protein, and so on. Six ‘healthy’ kidney specimens (sex- and age-matched) were taken from nephrectomies for cancer, and only normal, unaffected tissue portions (at least 2 cm from the cancer) were used for our analysis.

### Establishment of DKD mouse model

Male C57BL/6J mice (8 weeks, 25–30 g) were purchased from Beijing Viton Lihua Laboratory Animal Technology Co. Ltd, and placed in a controlled environment (temperature 23 ± 2 °C, humidity 50 ∼ 60%, light for 12 h, dark cycle for 12 h), with unlimited provision of standard mouse food and water. All experimental protocols were approved by the Experimental Animal Research Ethics Committee of Peking University School of Medicine. Anesthesia and euthanasia of mice are performed in full compliance with the American Veterinary Medical Association (AVMA) Animal Euthanasia Guidelines (2020).

To establish the DKD mouse model [33], mice were first left nephrectomized. Mice were anesthetized *via* intraperitoneal (i.p.) injection of 1% pentobarbital sodium (0.8 g/kg) diluted in sterile phosphate buffer solution (PBS) before surgery. One week later, the mice were given i.p. injections of 50 mg/kg streptozotocin (STZ) (cat. no. S0130; Beijing LABLEAD Business and Trade Co.) dissolved in 0.1 M sodium citrate buffer (pH 4.5) for 5 consecutive days. The blood glucose level one week after was measured using the glucometer system GA-3 (Sinocare Bio-Sensing Co.). Mice were considered to have diabetes when their blood glucose levels were > 16.7 mM after two consecutive determinations randomly. Mice were monitored for body weight and blood glucose. Mice were injected 1–2 units of insulin glargine subcutaneously when blood glucose was >20 mM. Mice were killed for blood and kidney collection after urine collection in metabolic cages at 6 weeks after STZ injection. Mice were euthanized using an overdose of sodium pentobarbital (>150 mg/kg) injected intraperitoneally, followed by adequate cardiac perfusion with PBS until the liver and kidneys lost their blood color before kidney collection. Sham mice (sex- and age-matched) were nephrectomized and received only sodium citrate buffer i.p. for 5 consecutive days. *n* = 6 mice per group.

### Hematoxylin–eosin (HE) and Masson staining

Mice kidney tissues were embedded in paraffin after being fixed in 10% formaldehyde at room temperature for 24 h and cut into 3‑µm‑thick sections. For general morphological assessment, Hematoxylin–Eosin (HE) staining was performed using HE Stain Kit (Beijing Solarbio Technology Co., Ltd.) according to the standard protocol provided by the reagent manufacturer. For fibrosis degree assessment, Masson staining was performed using Masson’s Trichrome Stain Kit (Beijing Solarbio Technology Co., Ltd.) according to the standard protocol provided by the reagent manufacturer.

### Immunohistochemical staining (IHC) and quantification

Paraffin-embedded sections of 10% formaldehyde fixed kidney tissues from patients and mice (each tissue section was 3 μm thick) were air-dried at room temperature and then baked at 60 °C for 2 h. After a series of graded ethanol dewaxing and hydration, the sections were subjected to high-pressure thermal repair in 0.05 M Tris–EDTA (PH 9.0) for 3 min, cooled, and then incubated with 3% H_2_O_2_ solution for 10 min to eliminate endogenous peroxidase. The sections were incubated with 10% goat serum (cat. no. ZLI-9056; Beijing ZSGB-BIO Bio-technology Co., Ltd.) or rabbit serum (cat. no. ZLI-9025; Beijing ZSGB-BIO Bio-technology Co., Ltd.) for 30 min at room temperature and protected from light for blocking, and then incubated overnight at 4 °C with RP215 antibody (1:1,000; donated by Prof. Qiu Xiaoyan, Peking University, Beijing, China), goat antimouse IgG antibody (1:800; cat. no. A90-131A; Bethyl Laboratories, Inc.), Col IV antibody (1:500; cat. no. ab236640; Abcam Trading Co., Ltd.) and α-SMA antibody (1:2,000; cat. no. ab32575; Abcam Trading Co., Ltd.), and replaced primary antibodies with PBS as negative control, followed by incubation with undiluted horseradish peroxidase‑labelled secondary antibodies (cat. no. PV‑6001 and PV‑6002; both Beijing ZSGB-BIO Bio-technology Co., Ltd.) at room temperature for 30 min. Finally, the sections were color developed with 3,3′-diaminobenzidine (DAB) under a special microscope with the same primary antibody color development time. The quantification of immunoreactivity for RP215, antimouse IgG, Col IV, and α-SMA was performed by the positive area in the cortical tubulointerstitial compartment of the kidney cortex. Specifically, we first used H DAB color deconvolution through the ImageJ 1.53c program (National Institute of Health,), and color_2 was chosen to assess the positive area through the Image Pro Plus program 6.0 (not less than 10 tubulointerstitial compartment fields per section for human specimens, and 30 tubulointerstitial compartment fields per section for mouse specimens), and the results were expressed as percentages. All procedural analyses and groupings were performed with the observer blinded to the clinical indicators of the patients.

### Cell culture and treatment

The immortalized human proximal renal tubular epithelial cell line (HK-2) was purchased from American Type Culture Collection and cultured in RPMI 1640 (Gibco; Thermo Fisher Scientific, Inc.) containing 10% fetal bovine serum (cat. no. SV30208.01; Shanghai Cytiva Life Science & Technology Co.) and penicillin (10,000 Units/ml) – streptomycin liquid (10,000 μg/ml) (Gibco; Thermo Fisher Scientific, Inc.) with 5% CO2 at 37 °C for proliferation. siRNA targeting the constant region of the IgG heavy chain (siRNA-IgG) and control siRNA (siRNA-NC; both Suzhou GeneParma Co., Ltd.) were transfected into HK-2 cells using GP-transfect-Mate transfection reagent (Suzhou GeneParma Co., Ltd.) according to the standard procedure in the reagent instruction manual. The sequences of the siRNAs were: siRNA-IgG sense, AGUGCAAGGUCUCCAACAATT; siRNA-IgG antisense, UUGUUGGAGACCUUGCACUTT; siRNA-NC sense, UUCUCCGAACGUGUCACGUTT; siRNA-NC antisense, ACGUGACACGUUCGGAGAATT. The transfected HK-2 cells were cultured with 5.56 mM (normal glucose concentration, NG) and 30 mM (high glucose concentration, HG) medium, respectively, and RNA was detected after 24 h, and protein expression was detected after 48 h.

### Western blotting (WB)

HK‑2 cells were lysed with TSD lysis buffer [1% SDS, 50 mM Tris‑HCl (pH 7.5), 50 mM DTT] containing 1% protease inhibitor (cat. no. GRF101; Shanghai EpiZyme Biomedical Technology Co.) to prepare protein samples. After quantification of the protein concentration using a BCA Protein Assay Kit (cat. no. ZJ101; Shanghai EpiZyme Biomedical Technology Co.), protein samples (30 µg) were separated by 7.5% SDS‑PAGE, and then transferred to nitrocellulose membranes. The membranes were blocked with protein-free rapid sealing solution (cat. no. PS108P; Shanghai EpiZyme Biomedical Technology Co.) for 30 min at room temperature, and incubated with primary antibody at 4 °C overnight. The following primary antibodies were used: α-SMA antibody (1:2,000; cat. no. ab32575), FN antibody (1:1,000; cat. no. ab268022), Col IV antibody (1:1,000; cat. no. ab236640), TGF-β1 antibody (1:1,000; cat. no. ab215715), GAPDH antibody (1:1,000; cat. no. ab181602) (all from Abcam Trading Co., Ltd.) and RP215 monoclonal antibody (1:1,000; donated by Prof. Qiu Xiaoyan, Peking University, Beijing, China), which specifically identified a carbohydrate‑associated epitope on non‑B‑IgG. The membrane was then incubated with secondary antibody conjugated to HRP‑labelled goat anti‑rabbit IgG (cat. no. ZB-2301) or anti‑mouse IgG (cat. no. ZB-2305) (both 1:10,000; both Beijing ZSGB-BIO Bio-technology Co., Ltd.) at room temperature for 1 h. The protein signals were detected by ultra-sensitive chemiluminescence detection kit (cat. no. SQ201; Shanghai EpiZyme Biomedical Technology Co.) and captured using Tanon 5200Multi Imaging System. ImageJ 1.53c program (National Institute of Health) was used for semi‑quantification.

### Reverse transcription‑quantitative polymerase chain reaction (RT‑qPCR) analysis

Total RNA was extracted from HK‑2 cells using RaPure Total RNA Kit (cat. no. R4011-03; Guangzhou Magen Biotechnology Co.), and the RNA concentration was assessed using a NanoDrop spectrophotometer (NanoDrop; Thermo Fisher Scientific, Inc.). Subsequently, 2 µg total RNA was reverse‑transcribed to cDNA using the RevertAid First Strand cDNA Synthesis kit (cat. no. K1622; Thermo Fisher Scientific, Inc.). qPCR was conducted on Applied Biosystems QuantStudio 5 (Thermo Fisher Scientific, Inc.) using the QuantiNova SYBR PCR Mix Kit (QIAGEN Enterprise Management Co.). The thermocycling conditions were as follows: An initial denaturation step at 95 °C for 2 min, followed by 40 cycles, (denaturation at 95 °C for 5 s, annealing at 60 °C for 10 s), and a final extension step at 95 °C for 15 s, 60 °C for 1 min and 95 °C for 10 s. Table 1 lists the primers, with β-actin serving as an internal reference. The relative expression is calculated using 2^–ΔΔCt^.

**Table 1.** List of primers.

Genes	Forward primer (5′–3′)	Reverse primer (5′–3′)
IgG (H)	CAGGACTGGCTGAATGGC	GGCGTGGTCTTGTAGTTGTT
α-SMA (H)	GGCATTCACGAGACCACCTAC	CGACATGACGTTGTTGGCATAC
FN (H)	GAGAATAAGCTGTACCATCGCAA	CGACCACATAGGAAGTCCCAG
TGF-β1 (H)	GGCCAGATCCTGTCCAAGC	GTGGGTTTCCACCATTAGCAC
β-actin (H)	GAAGTGTGACGTGGACATCC	CCGATCCACACGGAGTACTT

### Statistical analysis

Statistical analysis was performed with SPSS 26.0 (IBM Corp.). For human and mice data, normally distributed data were expressed as mean ± standard deviation, and non-normally distributed data were expressed as medians and interquartile ranges. Cell studies were conducted at least in triplicate, and all data are presented as the mean ± SEM. Continuous variables were compared by using the *t* test, Mann–Whitney *U* test, or ANOVA, as appropriate. Correlations were assessed by linear regression analysis. Categorical variables were expressed as percentages, which were evaluated by chi-square test or the Fisher’s exact test. The related factors for RP215 high titer were evaluated by logistic regression analyses. A two-sided *P* value < 0.05 was considered statistically significant.

## Results

### RTEC-IgG expression is upregulated and related with tubulointerstitial fibrosis and kidney function in DKD patients

**Figure 1.**
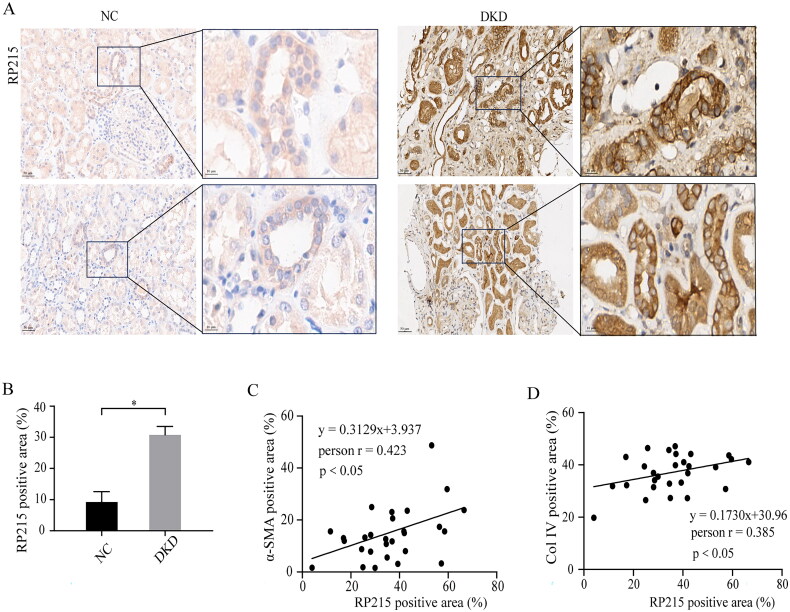
RTEC-IgG expression in renal tubulointerstitium and it’ s relation with EMT and TIF in DKD patients. RTEC-IgG expression (identified by RP215) was significantly elevated and positively correlated with EMT and TIF in DKD patients. (A and B) Immunohistochemistry staining for RTEC-IgG (identified by RP215) expression in renal tubulointerstitium of NC and DKD patients. Scale bars, 50 µm and 10 µm. (C) Correlation analysis between the RP215 and α-SMA positive area of DKD. (D) Correlation analysis between the RP215 and Col IV positive area of DKD. Data are presented as the mean ± SEM (NC, *n* = 6, DKD, *n* = 60). *p < 0.05 vs. NC. RTEC-IgG, IgG expressed by renal tubular epithelial cell; DKD, diabetic kidney disease; EMT, epithelial mesenchymal transition; TIF, tubulointerstitial fibrosis; NC, normal control.

RTEC-IgG (identified by RP215) was detected in kidney tissues from ‘healthy’ control (NC group) and DKD patients (DKD group) by immunohistochemistry as shown in Figure 1A and B. RTEC-IgG in the cytoplasm and cell membrane of renal tubular epithelial cells elevated significantly in patients with DKD compared with NC. Based on the results of RP215 staining in renal tubulointerstitium and using 12% of RP215 positive area as the cutoff value, the pathologic features of the patients with RP215 high titer (*n* = 44) and RP215 low titer (*n* = 16) at the time of kidney biopsy are shown in Table 2. Compared with the low titer group, patients with high titer had more serious interstitial fibrosis and tubular atrophy (*p* = 0.001) (Table 2). In addition, linear regression analysis showed a significant positive correlation between renal tubulointerstitial RP215 and α-SMA (*r* = 0.423, *p* < 0.05) (Figure 1C), as well as Col IV (*r* = 0.385, *p* < 0.05) (Figure 1D).

**Table 2.** Pathological features under light microscopy according to the renal tubulointerstitium RP215 staining.

Pathological lesions		All (*n* = 60)	Low titer (*n* = 16)	High titer (*n* = 44)	*P* value
Glomerular class (*n*%)	I	3 (5)	0 (0)	3 (6.82)	0.206
	II	9 (15)	1 (6.25)	8 (18.18)	
	III	47 (78.33)	14 (87.5)	33 (75)	
	IV	1 (1.67)	1 (6.25)	0 (0)	
IFTA score (*n*%)	0	0 (0)	0 (0)	0 (0)	0.001
	1	21 (35)	7 (43.75)	14 (31.81)	
	2	17 (28.33)	9 (56.25)	8 (18.18)	
	3	22 (36.67)	0 (0)	22 (50)	

IFTA, interstitial fibrosis and tubular atrophy.

The high titer of RP215 in renal tubulointerstitium was associated with more serious IFTA.

The clinical features based on RP215 staining in renal tubulointerstitium at the time of kidney biopsy are shown in Table 3. Compared with the low titer group, patients with high titer had lower estimated glomerular filtration rate (eGFR) (*p* = 0.045) and lower hemoglobin levels (*p* = 0.043) (Table 3). A cutoff value of eGFR 45 mL/min/1.73 m^2^ was used to distinguish between better and worse kidney function. Univariate logistic regression showed RP215 high titer in tubulointerstitium (OR, 12.5; 95% CI, 1.52, 103.04; *p* = 0.019), systolic blood pressure (SBP)>160 mmHg (OR, 4.16; 95% CI, 1.27, 13.58; *p* = 0.018), proteinuria > 3.5g/d (OR, 2.74; 95% CI, 0.84, 8.97; *p* = 0.095), hemoglobin (OR, 0.95; 95% CI, 0.92, 0.98; *p* = 0.002), and RAAS inhibitor application (OR, 0.2; 95% CI, 0.06, 0.68; *p* = 0.010) were associated with kidney function. Moreover, the multivariate logistic regression demonstrated that, in addition to SBP and hemoglobin, RP215 high titer in tubulointerstitium (OR, 9.43; 95% CI, 1.02, 87.1; *p* = 0.048) remained independently associated with worse kidney function (Table 4).

**Table 3.** Clinic features of DKD patients based on the renal tubulointerstitium RP215 staining.

Variables	All (*n* = 60)	Low titer (*n* = 16)	High titer (*n* = 44)	P value
Men (*n*%)	46 (76.67)	12 (75.00)	34 (77.27)	1.000
Age (years)	48.73 ± 11.72	46.75 ± 11.25	49.45 ± 11.93	0.434
Duration of diabetes (years)	10 (4.75, 16)	10 (3, 12.5)	10 (6, 18)	0.124
SBP (mmHg)	148.35 ± 22.51	145.12 ± 25.64	149.52 ± 21.46	0.508
DBP (mmHg)	86.5 ± 12.33	85.89 ± 12.87	87 ± 12.67	0.869
Diabetic retinopathy (*n*%)	38 (67.86)	9 (60.00)	29 (70.73)	0.661
HbA1c (%)	7.3 (6.5, 8.8)	8.1 (6.9, 9.7)	7.1 (6.4, 8.5)	0.134
Serum creatinine (μmol/L)	122 (96, 159)	101 (90, 139)	127 (96, 178)	0.062
eGFR (mL/min/1.73m^2^)	60.4 ± 28.52	71.36 ± 22.47	56.41 ± 29.64	0.045
Plasma albumin (g/L)	38.0 (32.5, 41.0)	35.8 (32.3, 39.1)	38.3 (33.6, 41.1)	0.457
24 h proteinuria (g/d)	4.12 (2.95, 9.31)	4.49 (2.73, 8.45)	4.60 (3.22, 9.27)	0.960
Urine erythrocyte (μl/l)	14 (6, 46)	33 (10, 72)	13 (6, 44)	0.379
Hemoglobin (g/L)	120 (104, 143)	134 (121, 144)	114 (99, 143)	0.043

SBP, systolic blood pressure; DBP, diastolic blood pressure; HbA1c, hemoglobin A1c; eGFR, estimated glomerular filtration rate.

The high titer of RP215 in renal tubulointerstitium is associated with lower eGFR and lower hemoglobin levels.

**Table 4.** Risk factor for worse kidney function by logistic regression analysis among DKD patients.

Factors	OR	95% CI	P
**Univariate**			
SBP > 160 mmHg (yes or no)	4.16	1.27–13.58	0.018
Proteinuria > 3.5g/day (yes or no)	2.74	0.84–8.97	0.095
Hemoglobin (g/L)	0.95	0.92–0.98	0.002
RAAS inhibitor (yes or no)	0.2	0.06–0.68	0.010
RP215 high titer in tubulointerstitium (yes or no)	12.5	1.52–103.04	0.019
Multivariate*			
SBP > 160 mmHg (yes or no)	4.54	1.08–19.02	0.039
Hemoglobin (g/L)	0.960	0.93–0.99	0.017
RP215 high titer in tubulointerstitium (yes or no)	9.43	1.02–87.1	0.048

SBP, systolic blood pressure; RAAS, renin–angiotensin–aldosterone system; OR, odds ratio.

As well as SBP > 160mmHg and hemoglobin, RP215 high titer in tubulointerstitium was independently associated with worse kidney function after *adjusted for gender, age, SBP, proteinuria, hemoglobin, and RAAS inhibitor.

There was no significant difference in the levels of serum IgG, and the use of statins or RAAS inhibitor in patients between RP215 high titer and lower titer (Table 5). Supplementary Table 1 summarized all statistically significant clinic and pathological features of DKD patients based on the renal tubulointerstitium RP215 staining.

**Table 5.** Serum immunoglobulins and therapies of DKD patients based on the renal tubulointerstitium RP215 staining.

Variables	All (*n* = 60)	Low titer (*n* = 16)	High titer (*n* = 44)	*P* value
Serum IgG (g/L)	10.42 ± 3.79	9.93 ± 4.25	10.6 ± 3.64	0.547
Serum IgA (g/L)	2.38 (1.6, 3.08)	2.48 (1.76, 3.27)	2.38 (1.55, 3.07)	0.453
Serum IgM (g/L)	0.9 (0.68, 1.2)	0.95 (0.82, 1.22)	0.9 (0.64, 1.19)	0.615
Serum IgE (IU/mL)	52.6 (19.0, 146.4)	84.1 (31.2, 119.5)	49.2 (16.8, 174.5)	0.759
Serum C3 (g/L)	1 ± 0.2	1.03 ± 0.22	0.99 ± 0.19	0.462
Serum C4 (g/L)	0.27 ± 0.08	0.26 ± 0.09	0.28 ± 0.07	0.515
Therapy				
RAAS inhibitor (*n*%)	44 (73.33)	14 (87.5)	30 (68.18)	0.243
Insulin (*n*%)	41 (68.33)	10 (62.5)	31 (70.45)	0.558
Statins (*n*%)	28 (46.67)	6 (37.5)	22 (50)	0.391

RAAS, renin–angiotensin–aldosterone system.

There was no significant difference in the levels of serum immunoglobulins and therapies in patients between RP215 high titer and lower titer.

### RTEC-IgG expression is upregulated in DKD mice and associated with tubulointerstitial fibrosis

**Figure 2.**
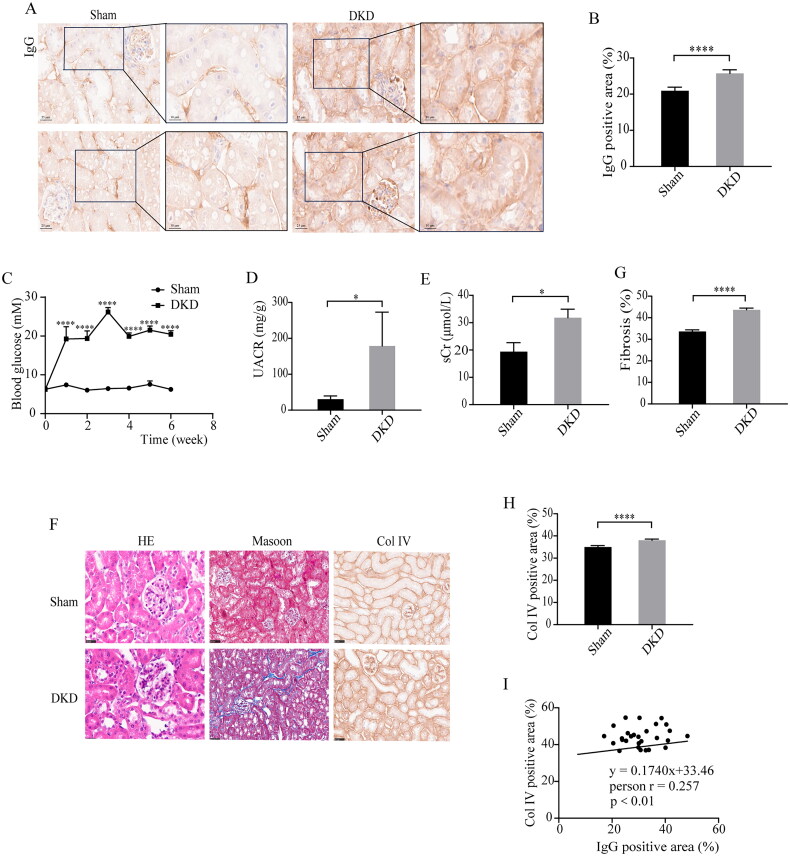
IgG Expression in renal tubular epithelial cells and it is relation with TIF in DKD model mice. IgG expression in renal tubular epithelial cells was significantly elevated and positively correlated with TIF in DKD model mice. (A and B) Immunohistochemistry staining for IgG expression in renal tubulointerstitium of Sham and DKD mice. Scale bars, 25 µm and 10 µm. (C) The blood glucose level in Sham and DKD mice. (D) Urine albumin to creatinine ratio (UACR) in Sham and DKD mice. (E) Serum creatinine (sCr) in Sham and DKD mice. (F) HE, Masson and immunohistochemistry staining of Col IV in renal tubulointerstitium from Sham and DKD mice. (G) Area of tubulointerstitial fibrosis of Sham and DKD mice assessed by Masson staining. (H) Immunohistochemistry analysis of Col IV expression in renal tubulointerstitium of Sham and DKD mice. (I) Correlation analysis between the IgG and Col IV positive area of DKD mice. Data are presented as the mean ± SEM. *n* = 6 mice per group. *p < 0.05, ****p < 0.0001 vs. Sham mice. RTEC-IgG, IgG expressed by renal tubular epithelial cell; DKD, diabetic kidney disease; TIF, tubulointerstitial fibrosis. HE, hematoxylin and eosin.

To further explore the role of RTEC-IgG in DKD, we established a DKD mouse model by unilateral nephrectomy combined with STZ treatment. The RTEC-IgG was also increased in mice with DKD compared with Sham (Figure 2A and B). One week after STZ injection, the random blood glucose levels of mice were over 16.7 mM (Figure 2C). Urinary albumin creatinine ratio (UACR) and serum creatinine (sCr) were higher in DKD mice than in Sham mice after six weeks of STZ injection (Figure 2D and E). HE staining showed proliferation of glomerular mesangial cells and stroma, and tubular dilatation and atrophy, and Masson staining and immunohistochemical staining for Col IV further confirmed collagen accumulation in the tubulointerstitium in DKD mice (Figure 2F–H). Further, linear regression analysis showed a significant positive correlation between tubulointerstitial IgG and Col IV (*r* = 0.257, *p* < 0.01) in DKD mice (Figure 2I).

### RTEC-IgG participates in the regulation of hyperglycemia-induced EMT and ECM deposition of RTECs via TGF-β1 signaling pathway

**Figure 3.**
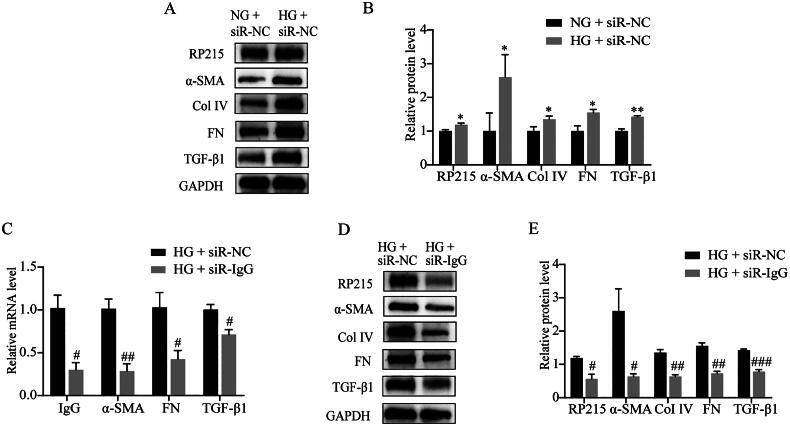
RTEC-IgG in the regulation of hyperglycemia-induced EMT and ECM deposition via TGF-β1 in HK-2 cells. α-SMA, Col IV, FN and TGF-β1, induced by high glucose, were accompanied by higher RTEC-IgG and were alleviated by knock-down of RTEC-IgG with siR-IgG in HK-2 cells. (A, B) Western blot analysis of the protein levels of RTEC-IgG (identified by RP215), α-SMA, Col IV, FN and TGF-β1 in NG or HG after siR-NC. (C) qPCR analysis of the mRNA levels of IgG, α-SMA, FN and TGF-β1 in HG after siR-NC or siR-IgG. (D, E) Western blot analysis of the protein levels of RTEC-IgG (identified by RP215), α-SMA, Col IV, FN and TGF-β1 in HG after siR-NC or siR-IgG. Data are presented as the mean ± SEM. *n* = 3. *p < 0.05 vs. NG + siR-NC. **p < 0.01 vs. NG + siR-NC. ^#^*p* < 0.05 vs. HG + siR-NC. ^##^*p* < 0.01 vs. HG + siR-NC. ^###^*p* < 0.001 vs. HG + siR-NC. NG + siR-NC, transfected with siR-NC and treated with 5.56 mM glucose. HG + siR-NC, transfected with siR-NC and treated with 30 mM glucose. HG + siR-IgG, transfected with siR-IgG and treated with 30 mM glucose. RTEC-IgG, IgG expressed by renal tubular epithelial cell; EMT, epithelial mesenchymal transition; ECM, extracellular matrix; NC, normal control.

Compared with normal glucose (5.56 mM), high glucose (30 mM) induced a significant overexpression of RTEC-IgG (identified by RP215) in HK-2 cells, and up-regulation of α-SMA, Col IV, FN and TGF-β1 expression (Figure 3A and B). Knockdown of RTEC-IgG by transfection of IgG-silencing siRNAs relieved the expression of α-SMA, Col IV, FN and TGF-β1 in HK-2 cells under high glucose (Figure 3C–E).

## Discussion

In this study, we found that RTEC-IgG was upregulated in patients and mice with DKD, which is pathologically associated with renal tubular EMT and TIF, and clinically associated with kidney function. High glucose induced a significant overexpression of RTEC-IgG in HK-2 cells, and knockdown of IgG expression relieved high glucose-induced TGF-β1 activation, tubular EMT and ECM deposition. To the best of our knowledge, this is the first time that the role of RTEC-IgG in DKD has been explored, and may provide new evidence for the pathogenesis of DKD and potential therapeutic targets for DKD.

Although it has been demonstrated that various diabetic substrates (including high glucose, glycosylation end products, and albumin), reactive oxygen species, angiotensin II, and TGF-β1 play important roles in EMT and TIF for patients with DKD, insufficient attention has been paid to the role of immunoglobulins in renal interstitial fibrosis of DKD. There were some researches that suggested that IgG along TBM was associated with tubulointerstitial damage [8], the source of IgG was believed ‘deposited’ from circulation. Inspired by discovery of non-B-Igs, our previous studies showed that RTECs could express IgG which can be specifically recognized by RP215 rather than commercial anti-IgG antibodies [28]. To found out the relationship between RTEC-IgG and tubular EMT and TIF as well as clinical features in patients with DKD, we first found that RTEC-IgG in patients with DKD can be specially bound by RP215, which is similar to non-B cells expressed IgG. The expression level of RTEC-IgG in the cytoplasm and cell membrane of RTECs, elevated significantly in patients with DKD, was positively correlated with tubular EMT and TIF, and the RP215 high titer is an independent risk factor for worse kidney function. We also found that RTEC-IgG was not parallel with serum IgG level, suggesting that the circulating IgG was not the sole source for ‘deposited’ IgG in the kidney.

To further explore the relationship between RTEC-IgG and TIF, we established a DKD mouse model by unilateral nephrectomy combined with STZ treatment. Compared with Sham mice, DKD mice showed higher blood glucose, UACR, sCr, and more severe glomerular mesangial matrix accumulation and tubulointerstitial fibrosis, similar to other studies of TIF in DKD using this mouse model [34, 35], suggesting that the DKD mouse model was successfully established. Because of the lack of antibodies specifically recognizing mouse non-B IgG, we tried to eliminate the effects of circulating IgG by performing full cardiac perfusion with PBS until the livers and kidneys were drained of blood prior to executing the mice. We found that IgG in the cytoplasm of RTECs elevated and is positively correlated with TIF in DKD mice.

High glucose induced the EMT and ECM deposition by activating the TGF-β1 signaling pathway in RTECs [4, 5]. IgG in adipose tissue activates macrophages via Ras signaling and consequently induces fibrosis through the TGF-β/SMAD pathway [24]. In this study, RTEC-IgG in HK-2 cells increased in high glucose, and were accompanied with TGF-β1 activation, tubular EMT and ECM deposition. Knockdown of IgG expression relieved TGF-β1 activation, tubular EMT and ECM deposition in high glucose state, suggesting that hyperglycemia induced RTEC-IgG expression, which stimulated the EMT and ECM deposition by activating TGF-β1. As a result, There is a potential for developing anti-DKD progression therapeutics targeting the RTEC-IgG, which could exert an anti-fibrosis effect through blocking the TGF-β1 signaling pathway.

Our work has some limitations. Firstly, it was a retrospective cohort study performed at a single center in China, so selection bias is inevitable and may not fully reflect the characteristics of the wider population and affect the generalizability of the results. Future studies with larger sample sizes and multiple centers are necessary. In this study, we did not find that proteinuria is an independent risk factor for worse kidney function after multivariate adjustment, which may be due to the biopsy inclusion condition with 4.12 g as the median of 24 h proteinuria, which may lead to the lack of early stage DKD patients. Secondly, we did not further explore the impact of RTEC-IgG high titer in tubulointerstitium on kidney outcomes, which can be achieved through continued follow-up. Finally, we did not isolate renal tubular cells from DKD mice for detecting IgG to adequately exclude the effect of circulating IgG.

In summary, our results suggest that RTEC-IgG is involved in the tubular EMT and interstitial fibrosis in DKD via TGF-β1 signaling pathway. We have described for the first time the potential of RTEC-IgG as a biomarker for the progression of DKD, providing novel insights into the pathogenesis of DKD and therapeutic targets against tubulointerstitial fibrosis.

## Supplementary Material

Fig1.tif

Fig2.tif

Fig3.tif

Supplementary material 2.pdf

Supplementary material 1.tif

## Data Availability

The datasets generated during and/or analyzed during the current study are available from the corresponding author on reasonable request.
