# Supplementary figures and images for: IgG expressed by renal tubular epithelial cells in epithelial mesenchymal transformation and interstitial fibrosis in diabetic kidney disease
Source: Ren Fail. 2025 Feb 3;47(1):2458764. doi: 10.1080/0886022X.2025.2458764 (PMC11795750; doi:10.1080/0886022X.2025.2458764)

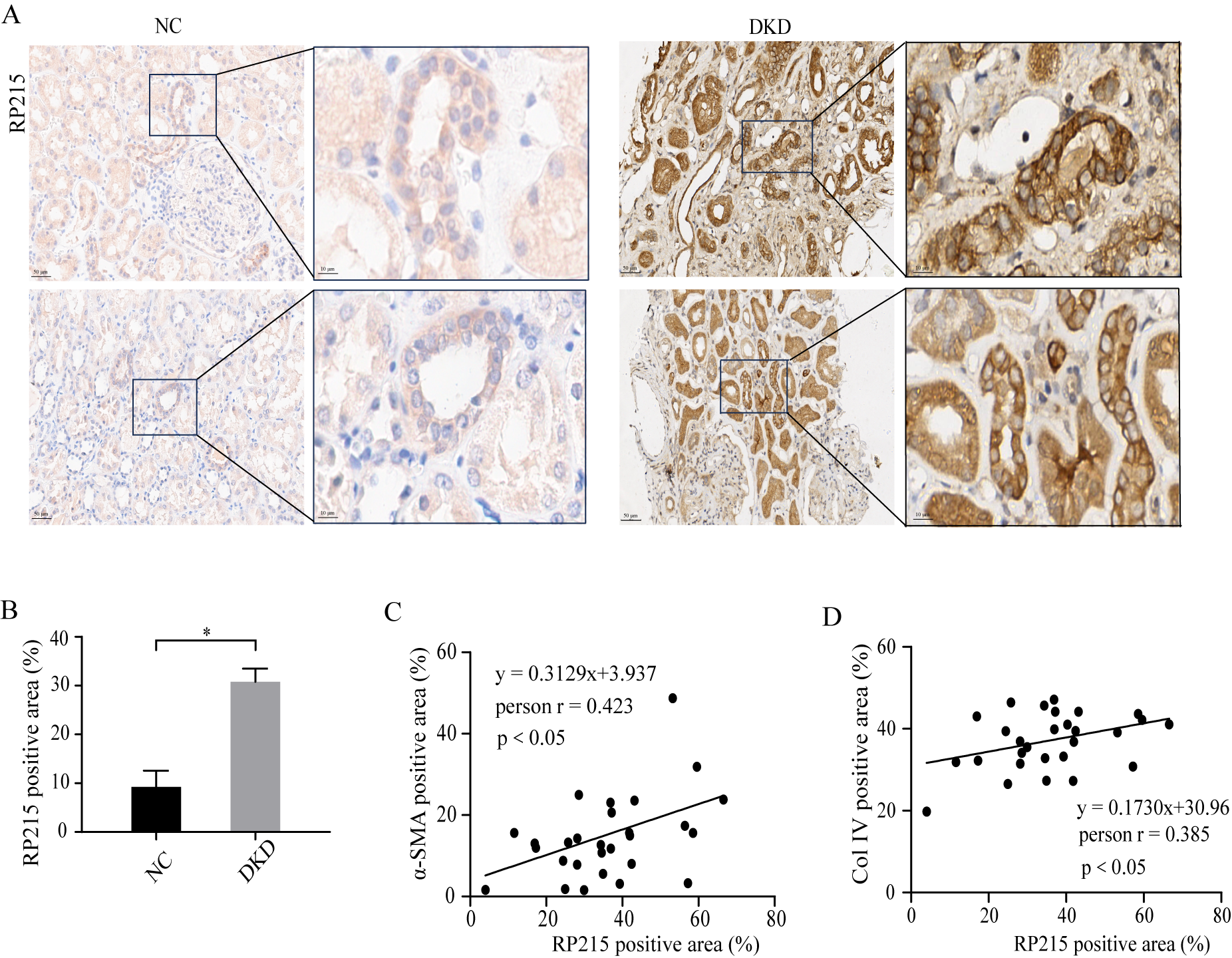

Supplement: Fig1.tif [file IRNF_A_2458764_SM5054.tif]

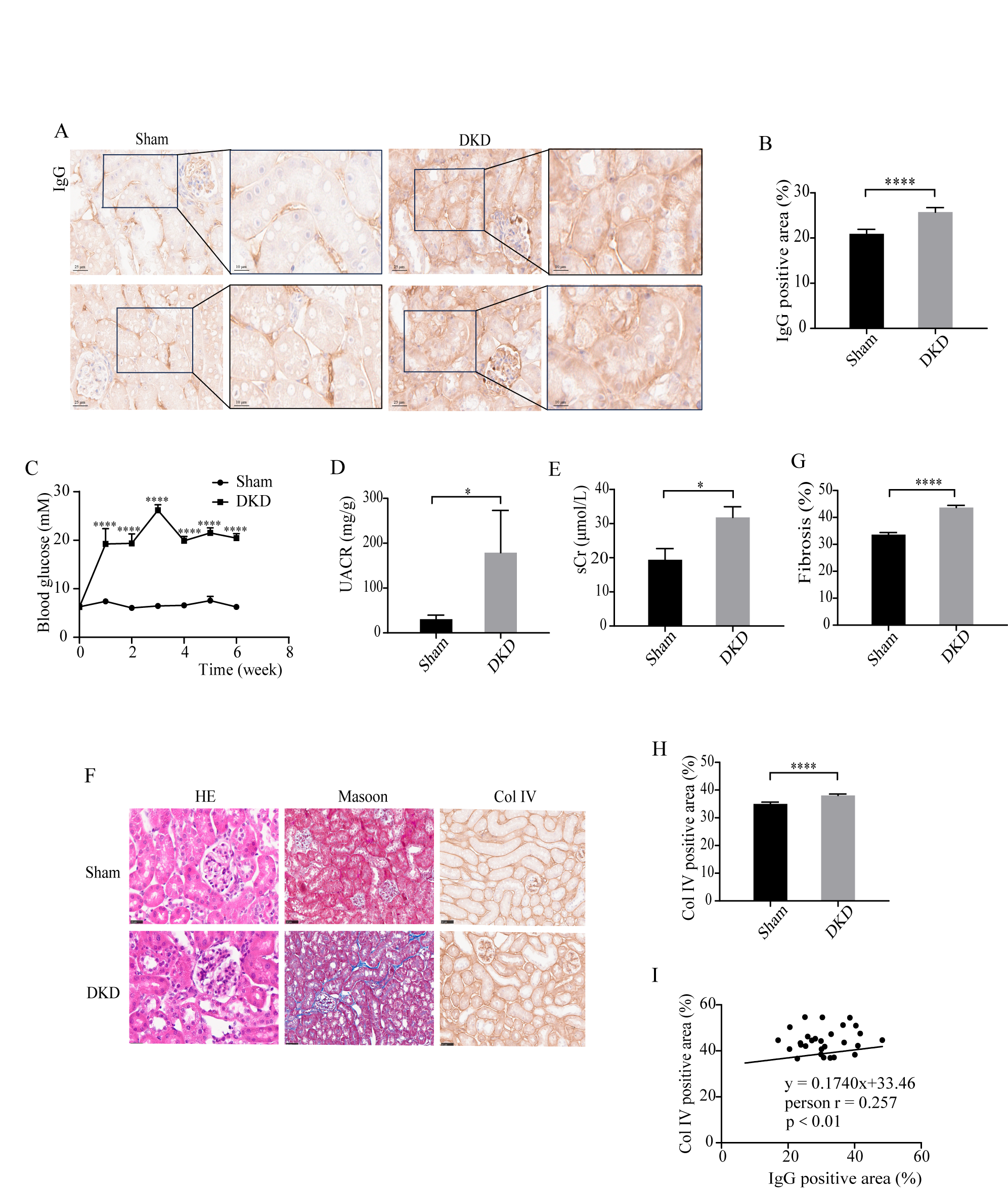

Supplement: Fig2.tif [file IRNF_A_2458764_SM5053.tif]

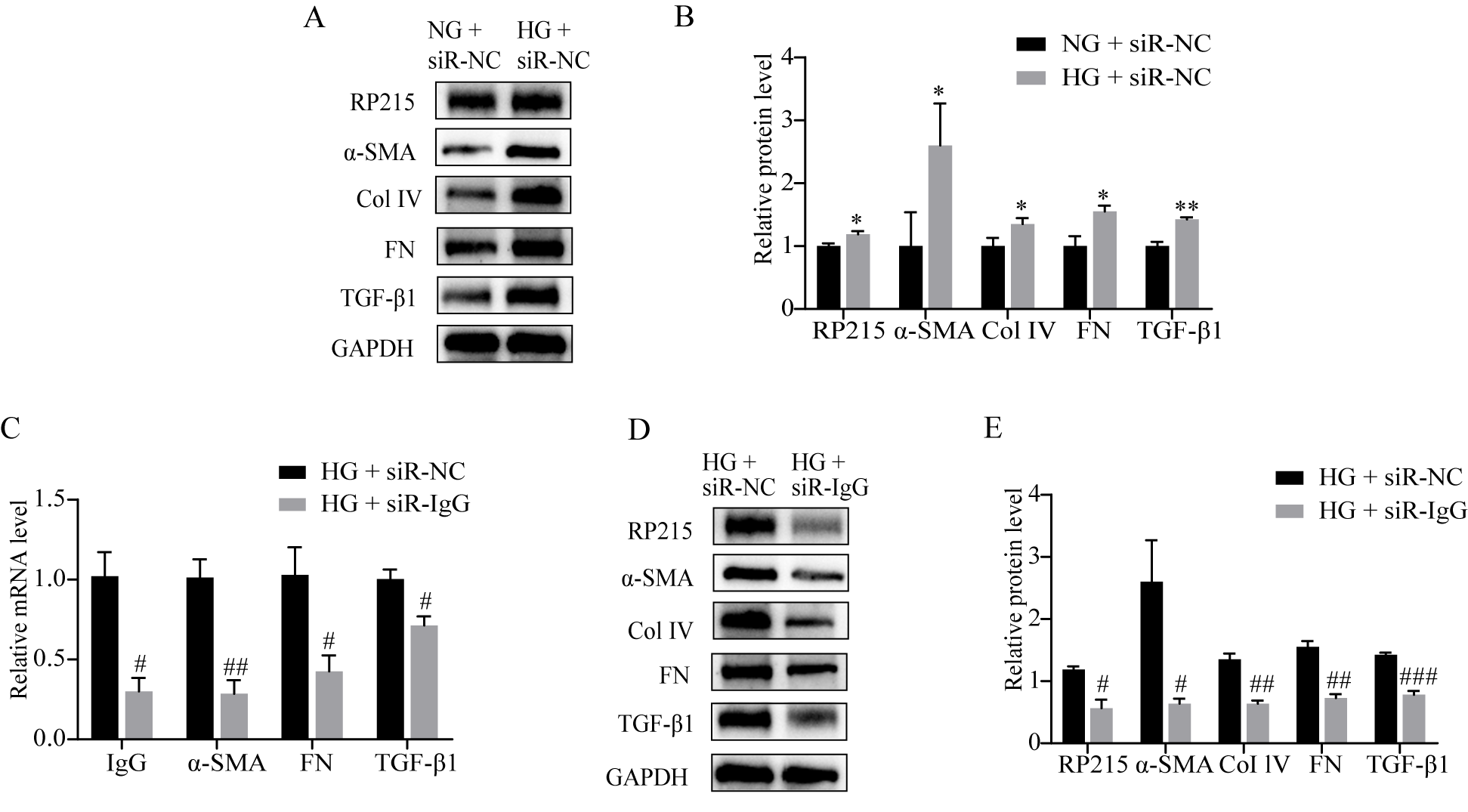

Supplement: Fig3.tif [file IRNF_A_2458764_SM5052.tif]

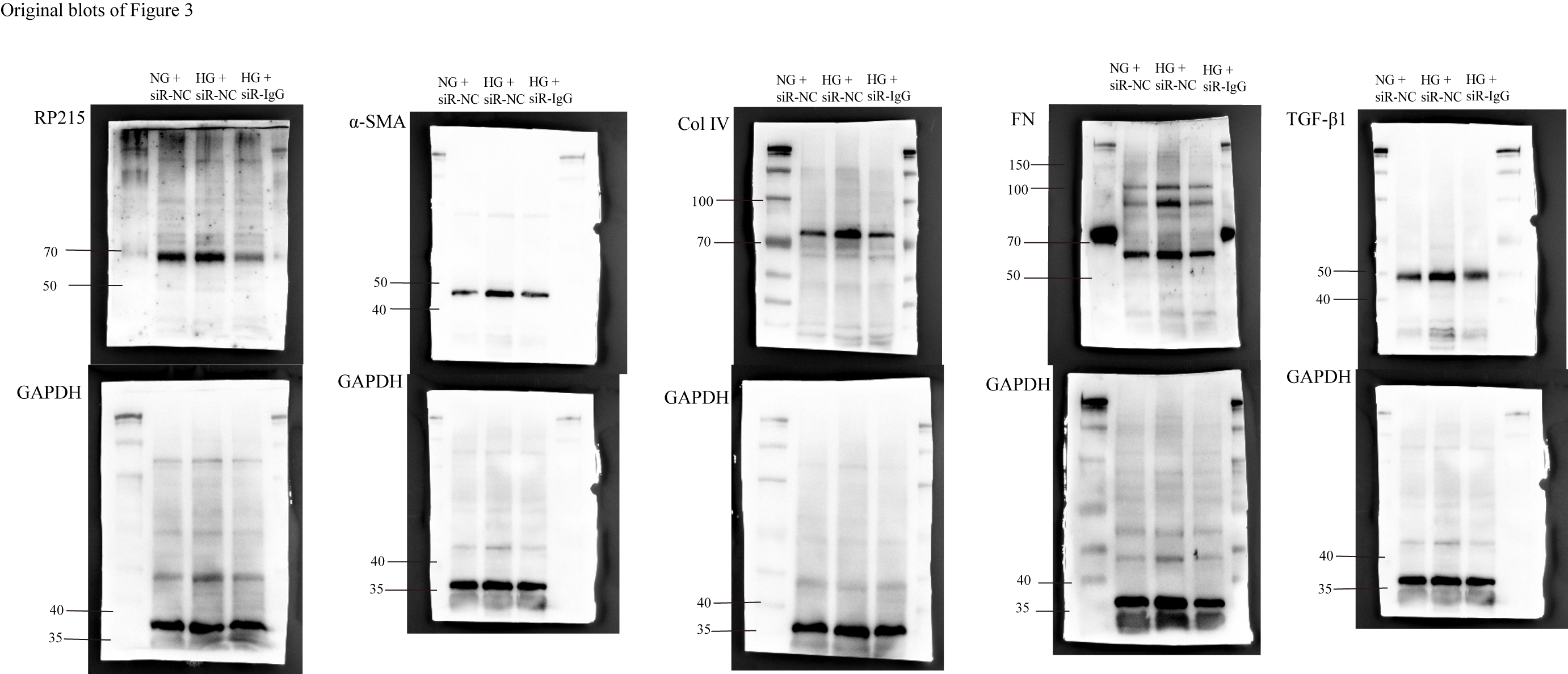

Supplement: Supplementary material 1.tif [file IRNF_A_2458764_SM5050.tif]
