# Supplementary material for: IgG expressed by renal tubular epithelial cells in epithelial mesenchymal transformation and interstitial fibrosis in diabetic kidney disease
Source: Ren Fail. 2025 Feb 3;47(1):2458764. doi: 10.1080/0886022X.2025.2458764 (PMC11795750; doi:10.1080/0886022X.2025.2458764)
Supplement: Supplementary material 2.pdf [file IRNF_A_2458764_SM5051.pdf]

北京大学第三医院医学科学研究伦理委员会

Peking University Third Hospital Medical Science Research Ethics Committee

学术伦理审查批件

Ethical Review Approval Notice

（2024）医伦审第（244-01）号

项目编号	IRB00006761-M2024248		
临床试验项目名称	近曲肾小管上皮细胞高表达IgG促进上皮细胞间充质转化和间质纤维化		
项目来源	在研课题		
试验类型	基础性研究		
产品名称	通用名：无	商品名：无	
药物注册分类		期别：	
CFDA药物临床试验批件号			
申办者/资助企业	无		
CRO公司	无		
临床试验单位及专业 /科室	北京大学第三医院/ 肾内科		
主要研究者及职称	王悦　　主任医师		
组长单位	无		
其他参加研究/合作单位（必要可附表）			
审查文件（必要可附表）	1.研究方案 2024年1月28日 V1.0<br>2. 生物样本、信息数据的来源证明 2024年1月28日 V1.0<br>3.信息保密说明 2024年1月28日 V1.0<br>4.知情同意书豁免申请 2024年1月28日 V1.0<br>5.研究成果发布形式说明 2024年1月28日 V1.0<br>6.研究者利益冲突说明 2024年1月28日 V1.0<br>7.再次签署知情同意书豁免申请 2024年1月28日 V1.0<br>8.研究材料诚信承诺书 2024年1月28日 V1.0<br>9.项目批准通知书		

（2024）医伦审第（244-01）号

投票结果	同意2票	作必要修改后同意0票	转会议审查0票
审查决定	同意		
审查声明	确认伦理委员会组成和执行符合根据中华人民共和国国家药品监督管理局联合国家卫生健康委员会颁布实施的《药物临床试验质量管理规范》、ICH-GCP、国家卫生健康委员会颁布实施的《涉及人的生物医学研究伦理审查办法》以及《赫尔辛基宣言》和国际医学科学组织委员会颁布的《人体生物医学研究国际道德指南》的伦理原则。<br>A statement confirming that the Ethics Committee is organized and operates according to Good Clinical Practice which is passed by NMPA and National Health Commission of the people’s Republic of China, ICH-GCP,Biomedical Research Ethics Review involving human which is passed by National Health Commission of the people’s Republic of China, Helsinki Declaration and ethical principles of International ethical guidelines for biomedical research involving human subjects which is passed by Council for International Organizations of Medical Science (CIOMS).		
审查意见	同意开展研究。		
跟踪审查频率	12 个月		
伦理审查批件有效期	2024年03月23日~2025年03月22日（请在有效期内启动实施，过期应重新申请审批）		
说明	1.本批件将在各研究中心及其伦理委员会备案。如果对方案在本机构的可行性（包括研究者的资格与经验、设备与条件等）有不同意见，请及时与本伦理委员会联系。<br>2.如对临床试验方案、知情同意书的任何修改，主要研究者更换，应及时通知伦理委员会，重新审查，获得批准后执行。<br>3.如发生严重不良事件以及影响研究风险受益比的非预期不良事件，研究者应在获知24小时内报告本伦理委员会。<br>4.暂停/提前终止/完成临床试验，请及时通知伦理委员会。<br>5.发现严重违反方案情况应及时报告伦理委员会。<br>6.完成临床试验后，请提交结题报告。<br>7.请按照批件要求的跟踪审查频率及时向伦理委员会递交跟踪审查报告。		
主任委员（签字）：[signature]	签发日期：2024-03-22	北京大学第三医院<br>医学科学研究伦理委员会（章）	

北京大学第三医院医学科学研究伦理委员会：北京市海淀区花园北路49号，邮编：100191
科研伦理综合办公室联系人：洪老师
联系电话：010-82265573
